# Comparative Assessment of Health Benefits of Praziquantel Treatment of Urogenital Schistosomiasis in Preschool and Primary School-Aged Children

**DOI:** 10.1155/2016/9162631

**Published:** 2016-08-18

**Authors:** Welcome M. Wami, Norman Nausch, Nicholas Midzi, Reggis Gwisai, Takafira Mduluza, Mark E. J. Woolhouse, Francisca Mutapi

**Affiliations:** ^1^Institute of Immunology and Infection Research, School of Biological Sciences, University of Edinburgh, Charlotte Auerbach Road, Edinburgh EH9 3FL, UK; ^2^Centre for Immunity, Infection and Evolution, School of Biological Sciences, University of Edinburgh, Charlotte Auerbach Road, Edinburgh EH9 3FL, UK; ^3^Department of Medical Microbiology, College of Health Sciences, University of Zimbabwe, P.O. Box A178, Avondale, Harare, Zimbabwe; ^4^Ministry of Health and Child Care, Murewa District Hospital, Murewa, Zimbabwe; ^5^Department of Biochemistry, University of Zimbabwe, P.O. Box MP167, Mount Pleasant, Harare, Zimbabwe; ^6^School of Laboratory Medicine and Medical Sciences, College of Health Science, University of KwaZulu-Natal, Durban 4000, South Africa

## Abstract

Schistosomiasis is a major public health problem in Africa. However, it is only recently that its burden has become recognised as a significant component impacting on the health and development of preschool-aged children. A longitudinal study was conducted in Zimbabwean children to determine the effect of single praziquantel treatment on* Schistosoma haematobium*-related morbidity markers: microhaematuria, proteinuria, and albuminuria. Changes in these indicators were compared in 1–5 years* versus* 6–10 years age groups to determine if treatment outcomes differed by age. Praziquantel was efficacious at reducing infection 12 weeks after treatment: cure rate = 94.6% (95% CI: 87.9–97.7%). Infection rates remained lower at 12 months after treatment compared to baseline in both age groups. Among treated children, the odds of morbidity at 12 weeks were significantly lower compared to baseline for proteinuria: odds ratio (OR) = 0.54 (95% CI: 0.31–0.95) and albuminuria: OR = 0.05 (95% CI: 0.02–0.14). Microhaematuria significantly reduced 12 months after treatment, and the effect of treatment did not differ by age group: OR = 0.97 (95% CI: 0.50–1.87). In conclusion, praziquantel treatment has health benefits in preschool-aged children exposed to* S. haematobium* and its efficacy on infection and morbidity is not age-dependent.

## 1. Introduction

Urogenital schistosomiasis, caused by the waterborne parasitic helminth* Schistosoma haematobium*, is an important but neglected tropical disease in Africa [1–3]. The disease causes significant paediatric health problems in endemic regions, with negative impacts on child health and development. In* S. haematobium* infections, damage caused by the parasite eggs lodged in tissues or exiting the body via the urine can result in bladder or urinary tract pathology, often characterized by blood in urine (macro- or microhaematuria), painful urination (dysuria), and proteinuria [4, 5]. High levels of albumin in urine have also been shown to be strongly correlated with urinary tract pathology due to* S. haematobium* infection [6]. Chronic infection with schistosomes in children is associated with complications such as anaemia, malnutrition, growth retardation, reduced physical fitness, and impaired memory and cognition [7, 8]. Infection and morbidity are controlled by treatment of infected individuals with the antihelminthic drug, praziquantel (PZQ) [9, 10]. Delay or a lack of intervention can result in more severe and irreversible forms of morbidity including urinary bladder cancer (squamous cell carcinoma) and chronic kidney disease, which may eventually result in death [11–13].

In several countries implementing schistosomiasis control programmes, the control strategies follow the directive by the World Health Assembly resolutions (WHA 54.19 and WHA 65.21) in 2001 [9], involving regular school based deworming using PZQ, aimed at reducing morbidity and promoting child health. However, a growing number of studies from Africa have shown that preschool children (aged ≤5 years) are also at high risk of schistosomiasis through passive exposure to infection whilst being bathed with infested water [14–18]. Furthermore, recent studies have also shown that PZQ treatment of schistosome infection is safe in preschool children [16, 19]. These findings have led to a new major recommendation by the World Health Organization (WHO) in 2010, stating that preschool-aged children should be considered for treatment through the regular health services and in ongoing public health intervention programmes [20]. This recent development in schistosomiasis control policy has heightened the need for a clear understanding on the health benefits of PZQ beyond the immediate reduction in infection levels in preschool-aged children in order to improve the effectiveness of interventions targeting this age group [21].

In a previous study in this population, we showed that, based on attributable fractions, albuminuria measured as the urine albumin-to-creatinine ratio (UACR) was the most reliable tool for detecting schistosome-related morbidity, followed by proteinuria and microhaematuria determined by dipsticks, visual urine inspection, questionnaires, and lastly clinical examination [22]. Thus, in this current study we focused on albuminuria, proteinuria, and microhaematuria. The aim of this study was to evaluate the immediate health benefits of PZQ in preschool-aged (1–5 years) children endemically exposed to* S. haematobium* at 12-week posttreatment and to determine if a single dose of PZQ (40 mg/kg) has sustainable impact on the health status of these children by assessing changes in infection rates and levels of schistosome-related morbidity markers (microhaematuria, proteinuria, and albuminuria) 12 months after treatment. Furthermore, we sought to determine whether the impact of PZQ treatment outcome is age-dependent by comparing these indicators in preschool and primary school-aged (6–10 years) children in the same population. The findings of our present study provide an operational recommendation for future studies on the control of paediatric schistosomiasis, whilst also giving further insights into the health benefits of antihelminthic treatment in preschool-aged children.

## 2. Materials and Methods

### 2.1. Ethics Statement and Consent

Prior to commencing the study, ethical clearance was obtained from the Medical Research Council of Zimbabwe (Approval Reference MRCZ/A/1615). In addition, the study received institutional approval from the University of Zimbabwe. Permission to conduct the study was obtained from the Provincial Medical Director, the District Educational Officer, and heads of schools in the study area. Study objectives and procedures were fully explained to the community, parents/guardians, teachers, and primary school-aged children in the local language, Shona. Samples were collected only after obtaining informed written parental consent and study participants' oral assent. Participants were permitted to withdraw from the study at any point without further obligation.

### 2.2. Study Site

The study was undertaken in Murewa district, in the northeast of Zimbabwe (31°90′E; 17°63′S) where* S. haematobium* is endemic. The area has low transmission of* S. mansoni *and soil transmitted helminths (STH) as previously reported in other studies [23, 24]. In a nationwide survey among school-aged children in 2010 in Zimbabwe, Midzi et al. [25] reported a schistosomiasis prevalence of 31.2% in Murewa district based on parasitological diagnostic methods. To confirm this reported level of schistosome infection in the community in our study site, we initially conducted a pilot study among primary school-going children (aged 6–10 years) as per sampling guidelines and recommendations by the WHO [26, 27]. A random sample of 50 compliant children from the two schools in the study area (25 from each school) was screened for schistosome infections by parasitology. Children found positive for infection during the pilot survey were treated with the recommended single dose of PZQ (40 mg/kg) but were then ineligible for participation in the main study.

### 2.3. Study Design and Population

**Figure 1:**
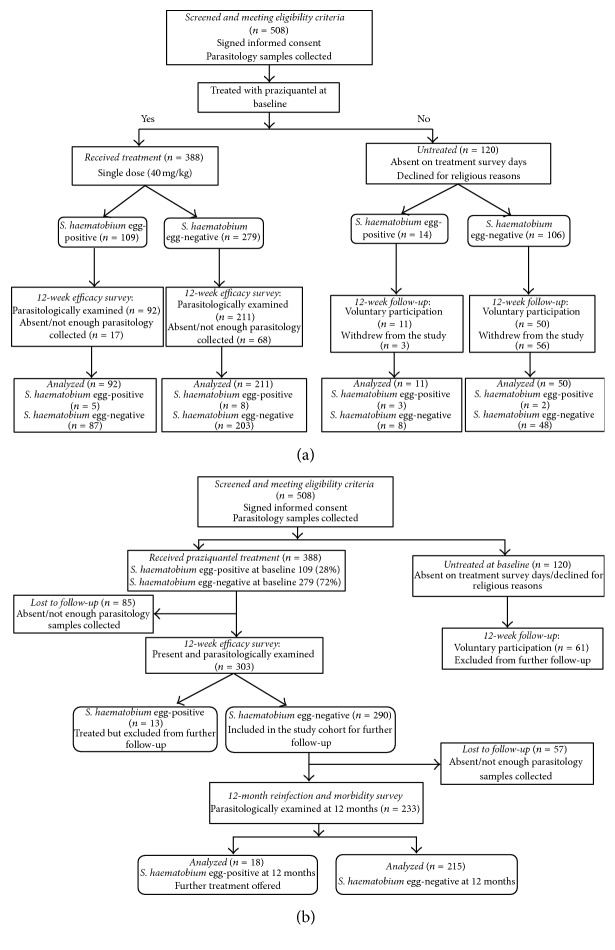
(a) Study design flowchart showing the number of children included for the final analysis to assess the effect of praziquantel treatment efficacy 12 weeks after treatment. Participants who preferred not to receive treatment but voluntarily remained in the study at 12 weeks were utilized as untreated controls. (b) Study design flowchart to assess the effect of praziquantel 12 months after treatment.

The longitudinal study was designed to relate changes in the levels of infection and markers of schistosome-related morbidity to the two age groups of children (1–5 years versus 6–10 years) conducted over a period of 12 months. There were two aspects to the longitudinal study design investigating (i) the short term effects of PZQ treatment which measured the morbidity markers and infection levels before treatment/baseline (February–March 2012) and 12 weeks (May 2012) after treatment, the workflow for this aspect of the study shown in Figure 1(a), and (ii) the longer-term effects of PZQ treatment which measured the morbidity markers and infection levels before treatment and 12 months (March 2013) after treatment; the workflow for this aspect of the study is shown in Figure 1(b). The 388 children who received treatment at baseline (Figures 1(a) and 1(b)) participated in both aspects of the study, so that the 303 children whose infection status was confirmed egg-negative at the 12-week efficacy check survey formed the treated cohort for follow-up at 12 months (Figure 1(b)). Untreated children (Figure 1(a)) were not followed up beyond 12 weeks. Children were recruited from two primary schools (typically for 6–10 years ages) and the early childhood development centres (ECDs) for preschool-aged children located within each of the primary schools. The schools also served as recruitment centres for children aged between 1 and 5 years not enrolled in any of the educational programmes in the study area who were also invited for enrolment to participate in this study. The villages within the study sites share the same river systems; therefore the transmission dynamics in the two schools (and associated ECDs) were similar.

### 2.4. Screening and Follow-Up Criteria

In order to be included in this study at baseline, children had to meet the following criteria: (i) they had been life-long (i.e., permanent) residents of the study area, (ii) they had no prior history of antihelminthic treatment (assessed by questionnaires administered to parents/guardians of all children), and (iii) they had provided at least two urine samples collected on consecutive days for the parasitological diagnosis of* S. haematobium* infection. For potential exclusion, children were assessed to identify (i) preexisting medical conditions or clinical symptoms of tuberculosis, any form of fever, or other signs of being unwell upon examination by study clinicians, (ii) recent major operation or illness as reported by parents/guardians, and (iii) infection with any of STHs or* S. mansoni*. No soil transmitted helminth infections were detected in this study cohort and 27 children positive for* S. mansoni *were offered PZQ treated but excluded based on this criterion. Participating children that were found egg-positive for* S. haematobium* at the 12-week efficacy check were treated with 40 mg/kg praziquantel but excluded from further follow-up to ensure that, in addition to new infections, only “true” reinfection was measured. Children who met the eligibility criteria, with informed consent, but would not accept treatment on religious grounds, or were absent on treatment survey days but voluntarily remained in our study cohort at 12 weeks effectively became untreated controls for evaluating the effect of treatment on schistosome-related morbidity markers. At the end of the study (12 months), PZQ treatment was offered to all children diagnosed egg-positive for infection.

### 2.5. Parasitological Methods

At all survey time points, urine samples were collected on three consecutive days for parasitological examinations.* S. haematobium* infection was detected by microscopic examination of the parasite eggs in urine, processed using the standard urine filtration method [28]. For each child, infection intensity was expressed as the arithmetic mean of egg counts per 10 mL urine of samples collected on consecutive days. At least two stool samples were also collected over three consecutive days, processed using the Kato-Katz method [29], and subsequently examined by microscopy for the diagnosis of* S. mansoni*. In a previous study we also determined the infection status of the children by serology; details of this methodology are described elsewhere [30].

### 2.6. Assessment of Morbidity Markers

Three urinary markers, microhaematuria, proteinuria, and albuminuria identified in our earlier published study [22], were chosen for investigating the effect of PZQ treatment on schistosome-related morbidity in this present study. Urine samples collected between 10:00 h and 14:00 h and processed on the first day of each survey time point were examined for microhaematuria and proteinuria, detected by dipstick reagent strip test (Uripath, Plasmatec, UK). Briefly, the reagent end of the test strip was dipped into fresh, well-mixed urine for 40 seconds. Upon removal, the test area was compared with a standard colour chart following the manufacturer's guidelines and the dipstick test results were calibrated as either positive or negative. To assess the stability of dipstick urinalysis [31], repeated tests (at least two repetitions) were performed on a random sample (*n* = 189) of urine specimens, allowing for time delay of up to 5 minutes between each of the repeated readings. There were no marked differences observed between repeated tests to suggest potential instabilities of urinalysis due to delays in dipstick testing. No observer bias was suggested by the urinalysis results when comparing the visually recorded dipstick readings to those read automatically using Siemens' CLINITEK Status + Analyzer (Bayer, UK). CLINITEK Microalbumin Reagent Strips (Bayer, UK) were used to determine the urine albumin-to-creatinine ratio (UACR) threshold levels (normal: UACR < 3.4 mg/mmol, abnormal: UACR ≥ 3.4 mg/mmol, or highly abnormal: UACR ≥ 33.9 mg/mmol), as read from the instrument following the manufacturer's guidelines. For each child, albuminuria as a marker of schistosome-related morbidity associated with urinary tract pathology was defined as a positive test for high abnormal UACR in each of the fresh urine samples examined [4].

### 2.7. Praziquantel Treatment

After collection of samples at baseline, compliant children were offered treatment with PZQ at the standard oral dosage of 40 mg/kg body weight. The PZQ drug was procured from Pharmaceutical and Chemical Distributors (Pvt) Ltd., Harare, Zimbabwe, a company registered and licensed to sell the antihelminthic drug in Zimbabwe. The tablets were swallowed with squash juice to reduce their bitter taste and a slice of bread to reduce the side effects of PZQ [16, 32]. For the very young children, the tablets were crushed according to the current recommendation by the WHO [20].

### 2.8. Sample Size Calculations

The relationship between schistosomiasis infection levels and indicators of morbidity is still unclear. We thus based our sample size calculations under the expectations that PZQ treatment reduces reinfection rates by more than 50% after 12 months. These expected effects are consistent with data from previous studies [16, 25] and are of sufficient magnitude to be of practical interest. Allowing for dropouts, our simulation studies using StatXact v8 (Cytel Software Corp., Cambridge, MA, USA) indicated that the sample sizes shown in Figures 1(a) and 1(b) will give us more than 80% power to detect age-related and treatment-related differences with significance level, *α* = 0.05.

### 2.9. Data Management and Statistical Analysis

Infection intensity was log-transformed: log_10_(egg count + 1) to meet the distributional assumptions of parametric test-statistics. Treatment efficacy against* S. haematobium* infection was assessed by means of cure rates (CR) and egg reduction rates (ERR), defined as CR = (number of children* S. haematobium* egg-negative at 12-week posttreatment/number of children confirmed egg-positive at baseline) × 100; ERR = (arithmetic mean egg count at baseline − arithmetic mean egg count at 12-week posttreatment/arithmetic mean egg count at baseline) × 100. The 95% confidence intervals (95% CI) for the ERR were calculated using a bootstrap resampling method with 1000 replicates in R 3.1.2 (R Development Core Team, Vienna, Austria), package = “eggCounts.” A Chi-square test (*χ* ^2^) or Fisher's exact test in the case of small expected frequencies (*n* < 5) was used for comparison of infection prevalence and cure rates between the two age groups. For infection intensity, a paired *t*-test was used to compare levels of infection before treatment versus after treatment. For each of the three urinary markers of schistosome-related morbidity (microhaematuria, proteinuria, and albuminuria), the outcome of interest was a dichotomous variable indicating whether the child presented with morbidity or not at a given time point (morbidity: 0 = negative; 1 = positive). The main predictor variables included the host factors sex (male versusfemale) and age group (1–5 years versus 6–10 years), treatment group (untreated versus PZQ treated), and time (pretreatment versus posttreatment). To assess the effect of treatment (pre/post) on morbidity markers, we used the method of generalized linear mixed model (GLMM) with a random effect to account for the correlation between children recruited from the same primary school/ECD as described earlier. The model-building process involved backward stepwise inclusion of the main effect covariate terms and their two-way interactions. All statistical analyses were performed using SAS 9.3 (SAS Institute Inc., Cary, NC, USA). The GLMMs were run using “PROC GLIMMIX” with a logit-link function to model the log odds of the probability of a child presenting with morbidity marker upon examination, and the parameter estimation was implemented using the method of penalized quasi-likelihood to account for overdispersion [33]. Comparisons for the binary morbidity marker responses between different subgroups of predictor variables were implemented using the contrast options within the “GLIMMIX” procedure. In all the analyses, multiple pairwise comparisons were adjusted for familywise type I error using the less conservative (i.e., has low rate of false negatives) simulation-based approach [34]. The level of significance was set at *P* < 0.05 for all the statistical tests performed. To aid the interpretation of the relative effect of treatment on infection levels and morbidity markers, standard errors (SE), 95% CI, and adjusted odds ratios (OR) were presented along with the significance test statistics.

## 3. Results

### 3.1. Demographic Characteristics and Pretreatment Infection Levels

Of the eligible children screened at baseline (see Figures 1(a) and 1(b)), a total of 508 children were included in the study, with 388 receiving PZQ treatment and 120 remaining untreated. Infection prevalence in the 508 children was 24.2% (95% CI: 20.5–28.0%), 6.7% (95% CI: 3.5–9.9%) in 1–5 years ages (*n* = 239), and 39.8% (95% CI: 33.9–45.7%) in 6–10 years ages (*n* = 269). In a subgroup of the 508 children, serological analysis showed that 63.0% (95% CI: 57.7–68.2%) of the children were seropositive for* S. haematobium* egg antigens, 39.8% (95% CI: 33.9–45.7%) were 1–5 years (*n* = 131), and 79.1% (95% CI: 73.4–84.8%) were 6–10 years (*n* = 201). The characteristics of the current study population are shown in Table 1. When considering the children treated at baseline and followed up at 12 weeks (*n* = 303, Figure 1(a)), the baseline infection prevalence determined by parasitology was 30.4% (95% CI: 25.2–35.6%) compared to 18.0% (95% CI: 8.1–28.0%) in the children who were not treated at baseline (*n* = 61, Figure 1(a)) but voluntarily remained in the study at 12 weeks.

### 3.2. Treatment Efficacy on Infection Levels at 12-Week Posttreatment

Twelve weeks after treatment, PZQ was efficacious at reducing* S. haematobium* infection among treated children in both age groups, as shown by high cure and egg reduction rates in Table 2. The overall cure and egg reduction rates in our study were 94.6% (95% CI: 87.9–97.7%) and 97.9% (90.6–99.5%), respectively. There was no significant difference (Fisher's exact test, *P* = 0.481) in cure rates between the two age groups (1–5 years versus 6–10 years) 12 weeks after treatment shown in Table 2.

### 3.3. Effect of Treatment on Infection/Reinfection Rates at 12-Month Posttreatment

Following initial treatment at baseline, children who had successful curative treatment were further followed up to determine the reinfection rates and proportion of new infections 12 months after intervention.  Table 3 shows the percentage proportion of treated children who were infected at baseline and then got reinfected (*n* = 7) and those newly infected (*n* = 11) within 12 months following treatment. The infection prevalence 12 months after treatment was low (i.e., <10% according to WHO classifications) as shown in Table 3. Furthermore, the proportion of infected/reinfected children did not significantly differ (*χ* ^2^ = 0.37; *P* = 0.542) between the two age groups: 1–5 years, *n* = 5: 6.3% (95% CI = 2.7–13.8%) versus 6–10 years, *n* = 13: 8.5% (95% CI: 4.8–14.4%). Similarly, the mean infection intensity level 12 months after treatment (mean = 0.74 egg/10 mL urine; SE = 0.27) was significantly lower (paired *t*-test = −7.95; *P* < 0.001) compared to the baseline level (mean = 14.3 egg/10 mL urine; SE = 4.63) in this study, with all egg-positive children carrying light infection intensities only.

### 3.4. Effect of Treatment on Morbidity Markers at 12-Week Posttreatment

**Figure 2:**
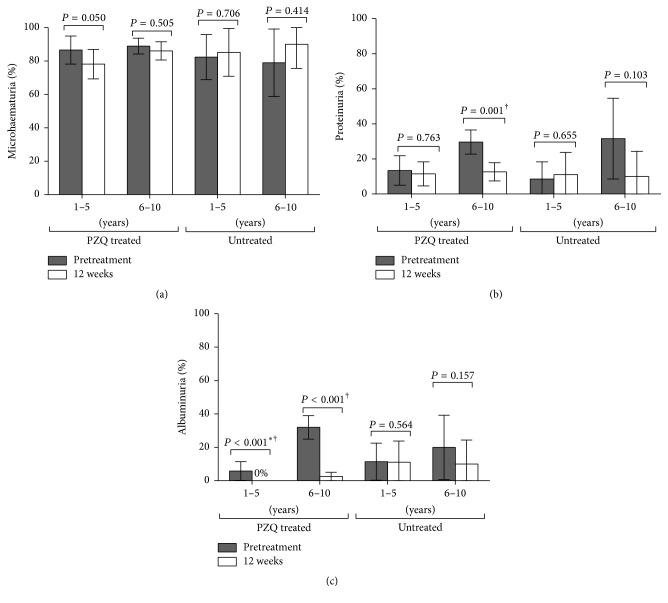
Effect of praziquantel (PZQ) treatment on the levels of urinary markers of schistosome-related morbidity 12 weeks after treatment. (a) Microhaematuria, (b) proteinuria, and (c) albuminuria. The error bars indicate the 95% confidence intervals. The *P* values for pairwise comparisons are from generalized linear mixed models investigating the probability of a child presenting with morbidity marker pre- versus posttreatment adjusted for sex and age group. Significant *P* values are marked with †. ^*∗*^Contrast  *P* value determined using the Binomial exact test.

The effect of PZQ on levels of morbidity markers was assessed in those children that had successfully cleared the infection at 12 weeks. Praziquantel significantly reduced levels of proteinuria and albuminuria 12 weeks after treatment (Figure 2). In addition, the odds of children who received praziquantel presenting with each of these markers of morbidity 12 weeks after treatment were lower compared to the odds before treatment, adjusting for sex and age group: proteinuria: OR = 0.54; (95% CI: 0.31–0.95) and albuminuria: OR = 0.05; (95% CI: 0.02–0.14). Furthermore, all the treated children aged 1–5 years successfully cleared albuminuria at 12 weeks and a significant reduction from baseline was observed among the 6–10 years ages (Figure 2). A mild decrease in the levels of microhaematuria among the treated preschool-aged children was also observed; however, these changes were not significant. Changes from baseline in the levels of the three markers of schistosome-related morbidity in untreated children at 12 weeks were not significant (Figure 2). Furthermore, when evaluating the overall changes in morbidity markers among the treated group (pre- versusposttreatment) compared to the changes in untreated children, it was observed that the odds of albuminuria were lower, OR = 0.43 (95% CI: 0.19–0.98; *P* = 0.045), adjusting for sex and age group.

### 3.5. Effect of Treatment on Morbidity Markers at 12-Month Posttreatment

**Figure 3:**
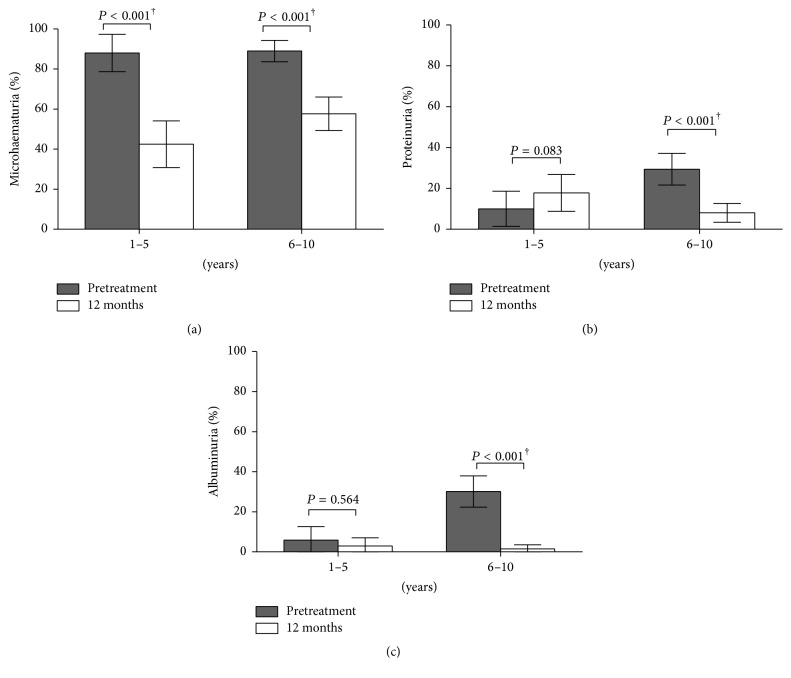
Effect of praziquantel treatment on levels of urinary markers of schistosome-related morbidity 12 months after treatment. (a) Microhaematuria, (b) proteinuria, and (c) albuminuria. Error bars indicate 95% CI and the *P* values for pairwise comparisons are from the generalized linear mixed models investigating the probability of a child presenting with morbidity markers before and after treatment, adjusted for sex and age group. Significant *P* values are marked with †.

Following curative treatment of infection at 12 weeks, as assessed by parasitology, children were further followed up and examined for morbidity at 12 months. Our analyses showed that when morbidity marker levels were investigated relative to baseline levels, microhaematuria significantly dropped in both age groups at 12 months after treatment (Figure 3). Furthermore, the results of GLMMs weighted by age group sample sizes showed that the odds of treated children presenting with microhaematuria 12 months after treatment did not significantly differ between children of 1–5 years and6–10 years: OR = 0.97; (95% CI: 0.50–1.87). In the case of proteinuria and albuminuria at 12 months, there was a significant reduction relative to baseline in the older age group, but not in 1–5 year ages (Figure 3). This was despite the initial reduction in albuminuria levels in this age group observed 12 weeks after treatment, as was shown in Figure 2.

## 4. Discussion

Praziquantel is currently the recommended antihelminthic drug of choice by the WHO for treating schistosomiasis and is effective against all the major schistosome species infecting humans [9]. As of present, the health benefits of PZQ treatment in children aged 5 years and below have not yet been extensively evaluated. Thus, in this study we sought to determine if PZQ treatment improves the current health status of children aged 1–5 years. The pretreatment infection levels reported in this study confirmed that preschool-aged children are exposed to schistosome infection, in concurrence with findings from other recent studies [16, 30], and further support the premise that if left untreated, these children are at an increased risk of developing severe morbidity that may cause serious health consequences and negatively impact their future quality of life [18, 35, 36]. In this study there were few children aged between 1 and 5 years who were excreting parasite eggs, but the prevalence of 6.7% is typical in this age group. More importantly, we and others [17, 30] have shown that the widely utilized egg count diagnostic method greatly underestimates infection prevalence in young children. Indeed, in a seroprevalence study of a subgroup of the 508 children in this group, the seropositive rate for* S. haematobium* egg antigen was 39.8% in 1–5 years ages. Thus, it is important to investigate the effects of treatment on other markers with health implications other than just the egg counts.

Our study results at 12 weeks after chemotherapy showed that a single standard dosage of PZQ was efficacious against* S. haematobium* infection in both 1–5 years and 6–10 years ages. The ranges of cure and egg reduction rates observed in this study for both age groups are consistent with data reported in the literature that have also shown high PZQ treatment efficacy within six weeks after treatment [16, 37, 38]. More interestingly, our study further revealed that PZQ treatment was effective in reducing* S. haematobium* infection levels in preschool-aged children as was observed in their older counterparts (6–10 years ages), further supporting their inclusion in current schistosomiasis control programmes [20, 39].

Infection prevalence remained lower 12 months after treatment compared to baseline levels, and the proportion of infected/reinfected children did not significantly differ between the two age groups. In addition, our study showed significant reductions in mean* S. haematobium* egg counts 12 months after treatment compared to baseline. These results are in agreement with those of previous studies from different endemic areas that also reported a lower risk of* S. haematobium* reinfection after annual school-based treatment [40, 41], indicative of the benefit of treatment in the prevention of infection through reduced parasite transmission at population level [40]. This combination of findings on the benefits of PZQ chemotherapy against infection provides some strong support for the need for inclusion of preschool-aged children in ongoing schistosomiasis control programmes, in order to increase the effectiveness of coverage of those infected, also recently highlighted in a study by Garba et al. [1].

Since schistosome-related morbidity is cumulative and progressive [42], decrease in current morbidity can reduce the long-term schistosomiasis sequelae. At 12 weeks after the first treatment, there was a significant decrease in the levels of the morbidity markers proteinuria and albuminuria in children successfully treated for infection. Interestingly, the effects of treatment were found not to be age-related, with microalbuminuria completely reversed in preschool-aged children at 12 weeks. The study also revealed that the prevalence of morbidity, diagnosed by presence microhaematuria, declined slowly, with a significant reduction observed after 12-month posttreatment. Our findings on the immediate health benefits of PZQ treatment were further reinforced by the results of the untreated group, showing no significant change in the levels of markers of schistosome-related morbidity at the 12-week follow-up.

The results showing persistently high levels of microhaematuria 12 weeks after treatment differ from some published studies that reported a considerable drop in microhaematuria within eight weeks after PZQ treatment [5, 43, 44]. However, most of these studies focused on older school-aged children who may have developed chronic infection. The high sensitivity of dipstick reagent strips in detecting microhaematuria, as previously reported [45], could also be one possible reason for these findings in our study. Another possible explanation for this delayed decrease in microhaematuria may be that most of the observed microhaematuria in these children may have likely been due to other health conditions other than schistosome infection. In an earlier study of this population we showed that the proportion of microhaematuria attributable to schistosome infection was less than that of albuminuria and proteinuria [22]. These results therefore need to be interpreted with caution.

One of the main objectives of schistosomiasis control programmes in endemic areas is preventative chemotherapy to combat the development of severe morbidity [20]. Thus, more efforts are needed to ensure that the immediate health benefits of chemotherapy are sustained in the targeted populations for effective control [1]. The current study revealed that a single PZQ treatment had sustained effects on the reduction of schistosome-related morbidity, as indicated by the levels of urinary markers that remained low 12 months after treatment. Furthermore, it is interesting to note that both the preschool and primary-school age groups demonstrated improved health beneficial treatment effect in terms of reduced morbidity burden measured by microhaematuria 12 months following single treatment with PZQ. In view of the current observations showing no age-related differences in treatment efficacy, it is thus practically possible for control programmes in endemic areas targeting preschool-aged children to be implemented using the existing treatment strategies designated for school-aged children.

Nevertheless, our study has limitations that must be considered when interpreting these results. Firstly, participation of the controls was on a voluntary basis for ethical reasons, hence leading to a small sample size for this group. This could have introduced additional uncertainties in the levels of infection and schistosome-related morbidity markers leading to a potential bias in the effects of treatment reported in this study. To minimise the effects of this potential bias, a random effect was included in the statistical models to account for some of the uncertainty. Secondly, since the majority of the children carried light infections, the parasitological cure rates may have been overestimated. Nonetheless, it is reassuring that efficacy rates reported in our study were comparable with those observed from other previous epidemiological studies in the same age range [1, 16].

## 5. Conclusions

In this study, we have demonstrated that praziquantel treatment effectively reduces not only* S. haematobium* infection levels, but also the levels of related morbidity in both preschool and primary school-aged children, with the reduction in some morbidity markers recorded within 12 weeks of treatment being sustained over a period of one year. In conclusion, praziquantel treatment has immediate health benefits in preschool-aged children exposed to* S. haematobium*, and the effect of praziquantel on infection and morbidity measures is not age-dependent. These findings are important for practitioners, policy makers, and stakeholders involved in the control of schistosomiasis and timely because of the current global drive to address the health iniquity created by the paucity of information on the impact of praziquantel treatment on schistosome-related morbidity in children aged 5 years and below.

## Figures and Tables

**Table 1:** Demographic characteristics of the study cohort followed up at 12-week and 12-month posttreatment with complete parasitology data for *S. haematobium *infection.

Variable	Characteristic	Treated group		Untreated controls^a^
		Baseline/12 weeks	12 months	Baseline/12 weeks
Sample size	*n*	303	233	61
Sex	M/F	142/161	108/125	29/32
Age (years)	Mean age (range)	6.6 (1–10)	6.9 (1–10)	4.9 (1–10)
	Median	6	7	5
Age group, *n* (%)	1–5 years	109 (36%)	80 (34%)	40 (66%)
	6–10 years	194 (64%)	153 (66%)	21 (34%)

^a^The data on voluntary untreated controls was only collected at baseline and 12-week follow-up surveys.

**Table 2:** Treatment efficacy of a single dose of praziquantel (40 mg/kg) against *S. haematobium* infection by age group at 12-week posttreatment. SE: standard error; CR: cure rate; ERR: egg reduction rate, with 95% confidence intervals (95% CI).

Age group	Baseline (pretreatment)			12-week posttreatment		Treatment efficacy	
	Number (screened and treated)	Number (diagnosed positive)	Mean egg count (SE)	Number of cases cured	Mean egg count (SE)	CR (95% CI)	ERR^a^ (95% CI)
1–5 years	109	8	2.7 (1.79)	8	0.0 (—)	100.0% (67.7–100.0%)	100.0% (—)
6–10 years	194	84	28.8 (8.40)	79	0.6 (0.45)	94.0% (86.8–97.4%)	97.9% (89.9–99.5%)

^a^The 95% confidence intervals for the ERRs were calculated using a bootstrap resampling method with 1000 replicates.

**Table 3:** Levels of *S. haematobium* reinfection and new infection rates among treated children in the study 12 months following treatment with single dose of 40 mg/kg praziquantel. Percentage proportions, with 95% confidence intervals (95% CI) of children infected with *S. haematobium* detected by parasitology.

Infection group	Sample size (*n*)	12-month posttreatment *S. haematobium* infection/reinfection rate	
		Number (egg-positive)	Prevalence (95% CI)
Reinfections^a^	76	7	9.2% (4.5–17.8%)
New infections^b^	157	11	7.0% (4.0–12.1%)

^a^Egg-positive at baseline and reinfected 12 months after treatment.

^b^Uninfected at baseline and found egg-positive 12 months after treatment.

## References

[1] Garba A., Barkiré N., Djibo A. (2010). Schistosomiasis in infants and preschool-aged children: infection in a single *Schistosoma haematobium* and a mixed *S. haematobium*-*S. mansoni* foci of Niger. *Acta Tropica*.

[2] Samuels A. M., Matey E., Mwinzi P. N. M. (2012). Schistosoma mansoni morbidity among school-aged children: a SCORE Project in Kenya. *American Journal of Tropical Medicine and Hygiene*.

[3] Hotez P. J., Fenwick A. (2009). Schistosomiasis in Africa: an emerging tragedy in our new global health decade. *PLoS Neglected Tropical Diseases*.

[4] Russell Stothard J. R., Sousa-Figueiredo J. C., Simba Khamis I., Garba A., Rollinson D. (2009). Urinary schistosomiasis-associated morbidity in schoolchildren detected with urine albumin-to-creatinine ratio (UACR) reagent strips. *Journal of Pediatric Urology*.

[5] Kahama A. I., Odek A. E., Kihara R. W. (1999). Urine circulating soluble egg antigen in relation to egg counts, hematuria, and urinary tract pathology before and after treatment in children infected with *Schistosoma haematobium* in Kenya. *The American Journal of Tropical Medicine and Hygiene*.

[6] Rollinson D., Klinger E. V., Mgeni A. F., Khamis I. S., Stothard J. R. (2005). Urinary schistosomiasis on Zanzibar: application of two novel assays for the detection of excreted albumin and haemoglobin in urine. *Journal of Helminthology*.

[7] Shaw J. G., Friedman J. F. (2011). Iron deficiency anemia: focus on infectious diseases in lesser developed countries. *Anemia*.

[8] WHO (2011). *Haemoglobin Concentrations for the Diagnosis of Anaemia and Assessment of Severity*.

[9] WHO (2002). Prevention and control of schistosomiasis and soil-transmitted helminthiasis.

[10] Richter J. (2003). The impact of chemotherapy on morbidity due to schistosomiasis. *Acta Tropica*.

[11] King C. H., Dangerfield-Cha M. (2008). The unacknowledged impact of chronic schistosomiasis. *Chronic Illness*.

[12] Olveda D. U., Olveda R. M., McManus D. P. (2014). The chronic enteropathogenic disease schistosomiasis. *International Journal of Infectious Diseases*.

[13] Smith J. H., Christie J. D. (1986). The pathobiology of schistosoma haematobium infection in humans. *Human Pathology*.

[14] Bosompem K. M., Bentum I. A., Otchere J. (2004). Infant schistosomiasis in Ghana: a survey in an irrigation community. *Tropical Medicine & International Health*.

[15] Odogwu S. E., Ramamurthy N. K., Kabatereine N. B. (2006). *Schistosoma mansoni* in infants (aged <3 years) along the Ugandan shoreline of Lake Victoria. *Annals of Tropical Medicine and Parasitology*.

[16] Mutapi F., Rujeni N., Bourke C. (2011). Schistosoma haematobium treatment in 1–5 year old children: safety and efficacy of the antihelminthic drug praziquantel. *PLoS Neglected Tropical Diseases*.

[17] Stothard J. R., Sousa-Figuereido J. C., Betson M. (2011). Schistosoma mansoni infections in young children: when are schistosome antigens in urine, eggs in stool and antibodies to eggs first detectable?. *PLoS Neglected Tropical Diseases*.

[18] Dabo A., Badawi H. M., Bary B., Doumbo O. K. (2011). Urinary schistosomiasis among preschool-aged children in Sahelian rural communities in Mali. *Parasites and Vectors*.

[19] Sousa-Figueiredo J. C., Pleasant J., Day M. (2010). Treatment of intestinal schistosomiasis in Ugandan preschool children: best diagnosis, treatment efficacy and side-effects, and an extended praziquantel dosing pole. *International Health*.

[20] WHO (2011). Report of a meeting to review the results of studies on the treatment of Schistosomiasis in preschool-age children.

[21] Mutapi F. (2015). Changing policy and practice in the control of pediatric schistosomiasis. *Pediatrics*.

[22] Wami W. M., Nausch N., Midzi N. (2015). Identifying and evaluating field indicators of urogenital schistosomiasis-related morbidity in preschool-aged children. *PLoS Neglected Tropical Diseases*.

[23] Midzi N., Sangweme D., Zinyowera S. (2008). Efficacy and side effects of praziquantel treatment against *Schistosoma haematobium* infection among primary school children in Zimbabwe. *Transactions of the Royal Society of Tropical Medicine and Hygiene*.

[24] Reilly L., Magkrioti C., Mduluza T., Cavanagh D. R., Mutapi F. (2008). Effect of treating *Schistosoma haematobium* infection on *Plasmodium falciparum*-specific antibody responses. *BMC Infectious Diseases*.

[25] Midzi N., Mduluza T., Chimbari M. J. (2014). Distribution of schistosomiasis and soil transmitted helminthiasis in zimbabwe: towards a national plan of action for control and elimination. *PLoS Neglected Tropical Diseases*.

[26] Nagelkerke N. J. D., Borgdorff M. W., Kalisvaart N. A., Broekmans J. F. (2000). The design of multi-stage tuberculin surveys: some suggestions for sampling. *International Journal of Tuberculosis and Lung Disease*.

[27] WHO (2006). *Preventive chemotherapy in Human Helminthiasis. Coordinated use of Anthelminthic Drugs in Control Interventions: A Manual for Health Professionals and Programme Managers*.

[28] Mott K. E., Baltes R., Bambagha J., Baldassini B. (1982). Field studies of a reusable polyamide filter for detection of *Schistosoma haematobium* eggs by urine filtration. *Tropenmedizin und Parasitologie*.

[29] Katz N., Chaves A., Pellegrino J. (1972). A simple device for quantitative stool thick-smear technique in *Schistosomiasis mansoni*. *Revista do Instituto de Medicina Tropical de Sao Paulo*.

[30] Wami W. M., Nausch N., Bauer K. (2014). Comparing parasitological vs serological determination of *Schistosoma haematobium* infection prevalence in preschool and primary school-aged children: implications for control programmes. *Parasitology*.

[31] Froom P., Bieganiec B., Ehrenrich Z., Barak M. (2000). Stability of common analytes in urine refrigerated for 24 h before automated analysis by test strips. *Clinical Chemistry*.

[32] Sousa-Figueiredo J. C., Betson M., Atuhaire A. (2012). Performance and safety of praziquantel for treatment of intestinal schistosomiasis in infants and preschool children. *PLoS Neglected Tropical Diseases*.

[33] Molenberghs G., Verbeke G. (2005). *Models for Discrete Longitudinal Data*.

[34] Edwards D., Berry J. J. (1987). The efficiency of simulation-based multiple comparisons. *Biometrics*.

[35] Stothard J. R., Sousa-Figueiredo J. C., Betson M. (2011). Closing the praziquantel treatment gap: new steps in epidemiological monitoring and control of schistosomiasis in African infants and preschool-aged children. *Parasitology*.

[36] Botelho M. C., Machado A., Carvalho A. (2016). *Schistosoma haematobium* in Guinea-Bissau: unacknowledged morbidity due to a particularly neglected parasite in a particularly neglected country. *Parasitology Research*.

[37] Tchuenté L.-A. T., Shaw D. J., Polla L., Cioli D., Vercruysse J. (2004). Efficacy of praziquantel against *Schistosoma haematobium* infection in children. *The American Journal of Tropical Medicine and Hygiene*.

[38] Coulibaly J. T., N'Gbesso Y. K., Knopp S., Keiser J., N'Goran E. K., Utzinger J. (2012). Efficacy and safety of praziquantel in preschool-aged children in an area co-endemic for *Schistosoma mansoni* and *S. haematobium*. *PLoS Neglected Tropical Diseases*.

[39] Stothard J. R., Sousa-Figueiredo J. C., Navaratnam A. M. D. (2013). Advocacy, policies and practicalities of preventive chemotherapy campaigns for African children with schistosomiasis. *Expert Review of Anti-Infective Therapy*.

[40] King C. H. (2006). Long-term outcomes of school-based treatment for control of urinary schistosomiasis: a review of experience in Coast Province, Kenya. *Memorias do Instituto Oswaldo Cruz*.

[41] Ahmed A. M., Abbas H., Mansour F. A., Gasim G. I., Adam I. (2012). Schistosoma haematobium infections among schoolchildren in central Sudan one year after treatment with praziquantel. *Parasites & Vectors*.

[42] King C. H. (2007). Lifting the burden of schistosomiasis—defining elements of infection-associated disease and the benefits of antiparasite treatment. *The Journal of Infectious Diseases*.

[43] Sacko M., Magnussen P., Traorŕ M. (2009). The effect of single dose versus two doses of praziquantel on *Schistosoma haematobium* infection and pathology among school-aged children in Mali. *Parasitology*.

[44] Stete K., Krauth S. J., Coulibaly J. T. (2012). Dynamics of *Schistosoma haematobium* egg output and associated infection parameters following treatment with praziquantel in school-aged children. *Parasites & Vectors*.

[45] King C. H., Bertsch D. (2013). Meta-analysis of urine heme dipstick diagnosis of *Schistosoma haematobium* infection, including low-prevalence and previously-treated populations. *PLoS Neglected Tropical Diseases*.

